# Clinical genetic analysis of an adult polyglucosan body disease (APBD) family caused by the compound heterozygous variant of *GBE1* p.R156C and deletion exon 3-7

**DOI:** 10.3389/fgene.2025.1514610

**Published:** 2025-03-19

**Authors:** Juan Zhu, Hong-Ping Yu, Jing Zou, Yi-Wu Zhang, Xin-Qi Han, Zi-Yan Xu, Li Chen, Qian Chen, Mei-Zhu Gao, Li-Jun Xie, Xi-Kui Zhang, Jie-Wei Luo, Yun-Fei Li, Li Zhang

**Affiliations:** ^1^ The Second Affiliated Hospital, Fujian University of Traditional Chinese Medicine, Fuzhou, China; ^2^ Department of Traditional Chinese Medicine and Nephrology, Shengli Clinical Medical College of Fujian Medical University, Fujian Provincial Hospital, Fuzhou University Affiliated Provincial Hospital, Fuzhou, China; ^3^ Department of Neurology of Youxi County General Hospital, Sanming, China; ^4^ Department of Oncology of Zhangzhou Traditional Chinese Medicine Hospital, Zhangzhou, China; ^5^ Department of Neurology, Fuzhou University Affiliated Provincial Hospital, Fuzhou, China

**Keywords:** APBD, adult polyglucosan body disease, GBE1, glycogen storage disease, neurogenic bladder, autonomic dysfunction

## Abstract

**Introduction:**

Adult Polyglucosan Body Disease (APBD) is a rare, autosomal recessive neurodegenerative disorder that affects both the central and peripheral nervous systems. It is primarily caused by mutations in the Glycogen Branching Enzyme 1 (*GBE*1) gene. APBD is typically associated with Ashkenazi Jewish populations, though it can occur in other ethnic groups. This study aims to expand the phenotypic and genetic spectrum of APBD, particularly in non-Ashkenazi Jewish patients, and to identify atypical genetic alterations linked to the disease.

**Methods:**

A 57-year-old Chinese male (Ⅱ3) presented with a 4-year history of progressive bladder dysfunction, upper and lower motor neuron impairment, sensory loss, and lower limb weakness, leading to difficulty with gait. Genetic testing was performed to identify potential pathogenic variants in the *GBE*1 gene. A family assessment revealed a sister (Ⅱ5) with the same clinical features. Both patients underwent genetic analysis, which included sequencing and deletion analysis.

**Results:**

Genetic testing revealed that both affected individuals (Ⅱ3 and Ⅱ5) carried compound heterozygous variants in the *GBE*1 gene: c.466C>T (p.R156C) in exon 4 and a large deletion of exons 3–7. The two pathogenic variants co-segregated in the family, confirming the diagnosis of APBD in these individuals.

**Discussion:**

This case expands the phenotypic and genetic spectrum of APBD, particularly by documenting its occurrence in non-Ashkenazi Jewish patients. Additionally, the identification of atypical genetic alterations, such as the large deletion in *GBE*1, provides new insights into the genetic basis of the disease and may aid in understanding its broader clinical manifestations. These findings suggest the need for a broader genetic screening approach in APBD diagnosis, especially in diverse populations.

## Introduction

APBD is a slowly progressive metabolic disorder classified as a leukodystrophy, caused by pathogenic variants in the *GBE1* gene (Souza et al., 2021; Akman et al., 2015). The classical form of APBD is most commonly associated with the homozygous *GBE1* variant p.Tyr329Ser (c.986A>C), and is characterized by a triad of clinical features after the age of 40: neurogenic bladder dysfunction, spastic paraplegia, and axonal neuropathy, which manifest in approximately 90% of patients. In addition, around two-thirds of patients present with varying degrees of cognitive impairment, with severe cases often leading to dementia (Klein et al., 2004), executive dysfunction, and involvement of both upper and lower motor neurons (Mochel et al., 2012; Carvalho et al., 2021). Patients harboring non-p.Tyr329Ser variants in *GBE*1 may exhibit atypical phenotypes, including Alzheimer’s disease-like dementia, stroke-like episodes, brain failure, and a history of infantile liver disease. The *GBE1* gene encodes the glycogen branching enzyme (GBE), which plays a crucial role in the proper formation of branched glycogen. Pathogenic variants in this gene result in reduced enzymatic activity, leading to a decrease in glycogen branching and the accumulation of slender, insoluble polyglucosan bodies (PB). These PBs accumulate in the central and peripheral nervous systems, with deposits found in the cytoplasm of astrocytes and nerve axons in various regions such as the cerebral hemispheres, cerebellum, spinal cord, nerve roots, and brainstem. Additionally, PB deposits have been observed in other tissues, including the liver, kidney, lung, heart, and skeletal muscle, albeit to varying extents (Souza et al., 2021; Akman et al., 2015; Carvalho et al., 2021; Akman et al., 1993; Ubogu et al., 2005; Paradas et al., 2014; Schroder et al., 1993). Glycogen Storage Disease Type IV (GSD-IV) is another disorder caused by pathogenic biallelic variants in the *GBE1* gene. (Lossos et al., 1998). The clinical spectrum of GSD-IV is heterogeneous, with severe neurological and muscular manifestations that can present in the neonatal period, infancy, childhood, adolescence, and adulthood. In adults, the condition is referred to as APBD (Koeberl et al., 2024). GSD-IV has a broad spectrum of phenotypes, including perinatal muscle and congenital neuromuscular subtypes, both of which result in early infant death. Additionally, GSD-IV includes progressive and non-progressive liver subtypes, with children affected by neuromuscular forms typically succumbing to liver failure by the age of five (Williams and Clementson, 1991). While APBD is considered a rare disorder, it predominantly occurs in individuals of Ashkenazi Jewish descent, with the p.Tyr329Ser (c.986A>C) mutation being most frequently reported in homozygous form (Souza et al., 2021). However, in this study, we present a case of familial APBD aggregation in a non-Ashkenazi Chinese family. The proband (Ⅱ3) exhibited the typical clinical triad of APBD. Genetic analysis identified a compound heterozygous variant in the *GBE1* gene, consisting of c.466C>T (p.R156C) (chr3:81699036) and a deletion spanning exons 3–7(NM_000158.3:c.[314_992+1del];[466C>T]). This study aims to provide a comprehensive clinical and genetic analysis of this Chinese family with APBD, contributing to the expanding phenotypic and genetic spectrum of the disease. We also explore the functional consequences of the identified *GBE*1 protein alteration (p.R156C), advancing our understanding of its role in disease progression.

## Material and methods

### Clinical case description

The proband (Ⅱ3), a 57-year-old male, presented with a four-year history of progressive numbness and weakness in both lower limbs, accompanied by bladder and bowel control dysfunction. The proband’s sister (Ⅱ5) exhibited a similar clinical presentation. She is a 53-year-old female who has been experiencing progressive weakness in both lower limbs over the past 2 years. Her urination and defecation remain normal. Comprehensive clinical data were collected from the proband and relevant family members, including complete blood counts, biochemical profiles, and tumor markers. Additionally, brain MRI, spinal MRI, EEG, EMG, and neuropsychological assessments were conducted for the proband. This study was approved by the Medical Ethics Committee of Fujian Provincial Hospital, and written informed consent was obtained from all participants.

### Extraction of genomic DNA

Peripheral blood samples from the probands and their family members were collected in anticoagulant tubes containing EDTA. Genomic DNA was subsequently extracted following the protocol provided in the Qiagen DNA Blood Mini Kit (CAT # 51106, Qiagen Co., Ltd.). To ensure the quality of the DNA samples, three methods of assessment were employed: DNA concentration and purity were measured using a NanoDrop spectrophotometer, DNA integrity was evaluated via agarose gel electrophoresis, and DNA concentration was further confirmed using the Qubit fluorometer. These rigorously tested DNA samples were then used for subsequent gene sequencing analysis.

### NGS sequencing whole exon sequencing and pathogenic variant screening and interpretation

In this study, second-generation gene sequencing (NGS) was performed on the proband (Ⅱ3). Genomic DNA was fragmented, followed by end repair, adenine base addition, and adaptor ligation. After PCR amplification and purification, the genomic DNA library was successfully constructed. The exonic regions were captured using the Naonda NEXome Core Panel and Hybridization and Wash Kit. After PCR linear amplification and library quality assessment, the target region coverage reached 100%, with an average sequencing depth of 129.3×, and 99.5% of the target region had a coverage depth of 20×. Quality-controlled libraries were then sequenced using the DNBSEQ-T7 platform (PE150). Target genes included *GBE1, IGHMBP2, SCN9A, SETX, HIKESHI, DES, COL6A2, AGRN*, and others (Table 1). The sequencing data were processed using a rigorous bioinformatics pipeline. Sequencing fragments were aligned to the human reference genome (UCSC hg19) using BWA software, followed by sorting and deduplication using Picard and Samtools markdup. Single nucleotide variants (SNVs) and insertion-deletion variants (Indels) were identified using GATK software. Based on next-generation sequencing (NGS), copy number variations (CNVs) at the exonic level were analyzed using the ExomeDepth tool. The variant data were further analyzed with Exomiser software to screen for potentially pathogenic variants, following the American College of Medical Genetics and Genomics (ACMG) guidelines. PCR amplification and Sanger sequencing were performed to validate the upstream and downstream regions of the *GBE*1 c.466C>T (p.R156C), which alters arginine to cysteine at position 156. The regions were amplified using the following primers: F: TCC​ACA​GAT​GTG​CTG​ATG​GT and R: CGC​CTG​GCA​TAA​TGG​TAA​CT, with an annealing temperature of 60°C. Primers were synthesized by Our primers were synthesized by Wuhan Genecreate Co., Ltd.

**TABLE 1:** Genetic Variants Detection Target Genes.

Genetic Variants Detection Target Genes				
IGHMBP2	SCN9A	SETX	HIKESHI	DES
GBE1	COL6A2	AGRN	D2HGDH	NHLRC1
PCCA	BRAT1	PAH	VPS13B	CFH
ABCA7	DUOX2	FS	CTCF	

### Real-time fluorescence quantitative PCR (RT-qPCR) was used to detect the expression level of GEB1 RNA

RNA was extracted from 5 family members using the Tripure Isolation Reagent Kit (#11667165001, Roche, Switzerland), and RNA expression levels were analyzed via reverse transcription quantitative PCR (RT-qPCR). Two pairs of primers were designed for the sequences flanking the pathogenic variant (primer design is shown in Table 2). For reverse transcription, a 20 μL reaction system was prepared by combining 4 μL of 5× All-In-One RT Mix, 1 μg of RNA template, 1 μL of primer mix, and DEPC water to a final volume of 20 μL. The components were mixed thoroughly by vortexing and brief centrifugation, then subjected to the following thermal protocol: incubation at 37°C for 10 min, 55°C for 15 min, and 95°C for 3 min. After the reaction, the mixture was briefly centrifuged and stored at −20°C for long-term use.

**TABLE 2:** Primer design is shown in the following table.

Primer name	Primer sequence
GBE1 mutation site pre-qPCR F*	TCC​ACA​GAT​GTG​CTG​ATG​GT
GBE1 mutation site pre-qPCR R*	CCA​TGA​GGC​ACG​AGT​ACA​GA
qPCR after GBE1 mutation site F	ATA​AGT​CGC​TGG​CAT​TTT​GG
qPCR after GBE1mutation site R	CGC​CTG​GCA​TAA​TGG​TAA​CT
hGAPDH F	CAA​GGT​CAT​CCA​TGA​CAA​CTT​TG
hGAPDH R	GTC​CAC​CAC​CCT​GTT​GCT​GTA​G

*F as Forward and R as Revers.

The resulting cDNA from the reverse transcription step was diluted 20-fold and stored on ice for subsequent RT-qPCR analysis. A 20 μL RT-qPCR reaction system was prepared, consisting of 10 μL 2× Ultra SYBR Mixture, 2 μL of 0.2 μM primers (forward and reverse mix), and 8 μL of diluted cDNA. The thermal cycling conditions included a melting curve analysis, ranging from 65°C to 95°C, for 40 cycles. RNA samples were reverse transcribed to cDNA, which was then amplified by PCR, and the products were sent for sequencing. The primer sequences are shown in Table 3.

**TABLE 3:** PCR amplification primers.

Primer name	Primer sequence5′-3′
GBE1 PCR F	TCC​ACA​GAT​GTG​CTG​ATG​GT
GBE1 PCR R	CGC​CTG​GCA​TAA​TGG​TAA​CT

## Result

### Clinical phenotype

The proband (Ⅱ3), a 57-year-old male, first experienced unexplained numbness in both lower limbs at age 53, beginning in the soles of his feet and gradually progressing. This was accompanied by difficulty walking, characterized by strenuous leg lifting and an unstable gait, eventually requiring a wheelchair for mobility. Over the past 5 years, he has experienced progressive memory loss and bladder dysfunction, often leading to urinary incontinence and defecation difficulties. He also exhibits cognitive impairment, including forgetfulness, difficulty with executive function, and ataxia, affecting his ability to perform daily tasks. Notably, the proband does not report limb convulsions, personality changes, vision or hearing loss, slurred speech, or dysphagia. His medical history includes hypertension, impaired glucose tolerance, and hypertriglyceridemia. He denies alcohol consumption, drug use, or phenothiazine medications. There is no history of psychosis, encephalitis, brain injury, stroke, or vaccine-related adverse reactions. The proband’s parents (Ⅰ1,Ⅰ2) are not related, and neither has a similar medical history. Physical examination revealed a height of 172 cm, a weight of 60 kg, and a body mass index (BMI) of 21.26 kg/m^2^. Nutritional status was good, and blood pressure was normal in a supine position. Cardiopulmonary and abdominal examinations showed no abnormalities. Neurological examination indicated that the proband was alert, with clear speech, relevant responses, and cooperation during testing. Memory impairment was noted, with a Mini-Mental State Examination (MMSE) score of 27 and a Montreal Cognitive Assessment (MoCA) score of 21, both consistent with his high school education level. Cranial nerve examination revealed no abnormalities. Mild muscle atrophy was observed in the lower limbs, with muscle strength rated as 5 in the upper limbs, 4 in the proximal lower limbs, and 4+ in the toes. Tendon reflexes were symmetrical but absent in the limbs, and bilateral pathological signs were positive. Sensory examination revealed diminished pain perception below the rib level and in the forearms, along with reduced position sense in the feet. The hard eye closure sign was positive. The finger-to-nose test was normal, but the bilateral heel-knee-shin test indicated lack of cooperation. The neck was supple, and bilateral Kernig’s sign was negative. Auxiliary tests showed normal thyroid function, adrenocorticotropic hormone (ACTH), and cortisol levels from previous hospital evaluations. Biochemical tests, autoimmune markers, and tumor markers were within normal ranges. MRI scans of the brain, cervical spine, thoracic spine, and lumbar spine revealed extensive cerebral cortex and cerebellar atrophy, subcortical degeneration in the lateral frontal and parietal lobes, and white matter changes adjacent to the lateral ventricles. Additionally, spinal cord MRI indicated medulla oblongata and spinal cord atrophy. Electromyography (EMG) results are detailed in Supplementary Table S1.

**FIGURE 1:**
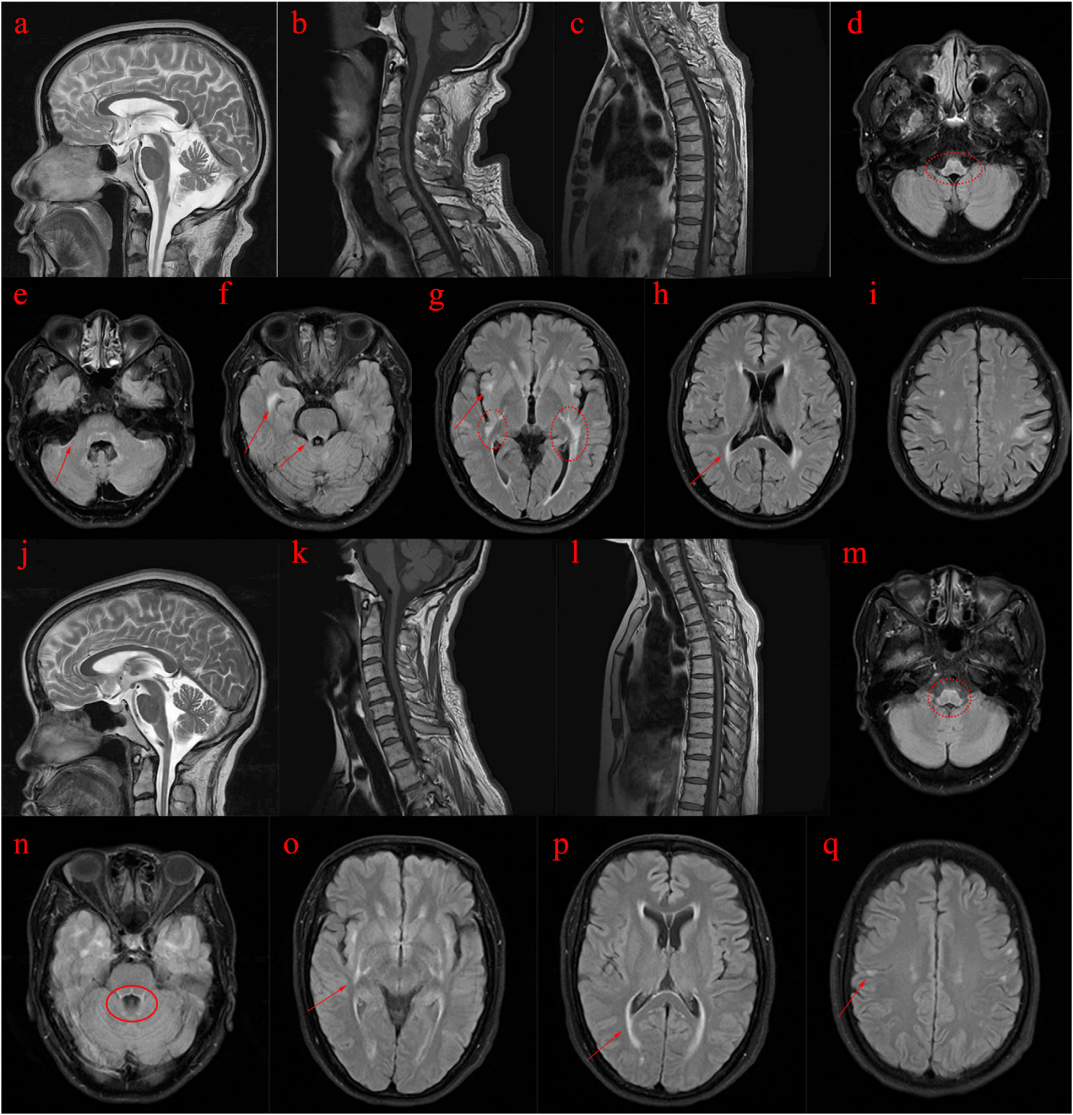
MRI images of the proband (II3) and his sister (II5). **(A–I)**: MRI scans of the proband (Ⅱ3) reveal abnormal spinal cord and brain structures. **(J–Q)**: MRI scans of the proband’s sister (Ⅱ5) also indicate abnormal spinal cord and brain structures. **(A–C)**: T2-weighted images show atrophy of the cerebral cortex and cerebellum, while T1-weighted images depict pronounced atrophy in the cervical and thoracic spinal cord. **(D–I)**: T2 FLAIR images demonstrate abnormal high signal intensity in the white matter with a symmetrical pattern, primarily affecting the pyramidal tract, periventricular regions, temporal lobe, posterior limbs of the external capsule, internal capsule, and surrounding the brainstem. **(J–L)**: T2-weighted images of the proband’s sister reveal cerebral and cerebellar atrophy, along with significant atrophy of the cervical and thoracic spinal cord on T1-weighted images. **(M–Q)**: T2 FLAIR images show abnormal high signal intensity in the white matter, symmetrically affecting regions similar to those in the proband, including the pyramidal tract, periventricular area, temporal lobe, external and internal capsule, and around the brainstem.

The proband’s sister (Ⅱ5), aged 53, began experiencing lower limb weakness and chills at age 51. She also had frequent falls but maintained normal bowel function and memory. Physical examination revealed no abnormalities in the heart, chest, or abdomen. She was conscious but had mildly slurred speech, scoring 27 on the MMSE and 19 on the MoCA (consistent with a junior high school education level). No abnormalities were noted in the remaining cranial nerves. No muscle atrophy was observed, but muscle tone was slightly increased. Muscle strength was graded 5 in the upper limbs and 5- in the lower limbs. Sensory function was normal, and tendon reflexes were present (+), with bilateral pathological signs (+). MRI scans of the brain and spinal cord in both the proband and his sister showed cortical, cerebellar, and spinal cord atrophy, along with multiple symmetrical white matter changes (Figure 1). Table 4 summarizes the clinical features of the proband and his sister.

**TABLE 4:** Summary of the Clinical Presentations of Proband II3 and His Sister II5.

	Ⅱ3	Ⅱ5
Age at diagnosis	53	51
Sex	man	female
Gait disorder	+	+
Auxiliary tool	wheelchair	−
Bladder dysfunction	+	+
Paresthesia	+	+
Dystonia	+	+
Muscle atrophy	+	−
Hypertonia	+	+
Ataxia	−	−
Cognitive impairment	Mild	−
MMSE	27	27
MoCA	21	19
White matter abnormality	+	+
Cerebellar atrophy	+	+
Spinal cord atrophy	+	+

### Whole exome sequencing (WES), copy number variation (CNV) and mitochondrial DNA full-length sequencing

This c.466C>T substitution involves the substitution of cytosine with thymine, resulting in a change of arginine (Arg) at position 156 to cysteine (Cys) in the *GBE1* protein, which ultimately leads to a structural alteration of the protein. The p.R156C mutation was inherited from the mother (I2). Family screening revealed that individuals II3, II5, II9, III1, III3, and III5 are carriers of the c.466C>T (p.R156C) heterozygous mutation. Copy number variation (CNV) analysis revealed a deletion of exons 3–7 (CN = 1), inherited from the father (I1).

### RT-qPCR results of GEB1

**FIGURE 2:**
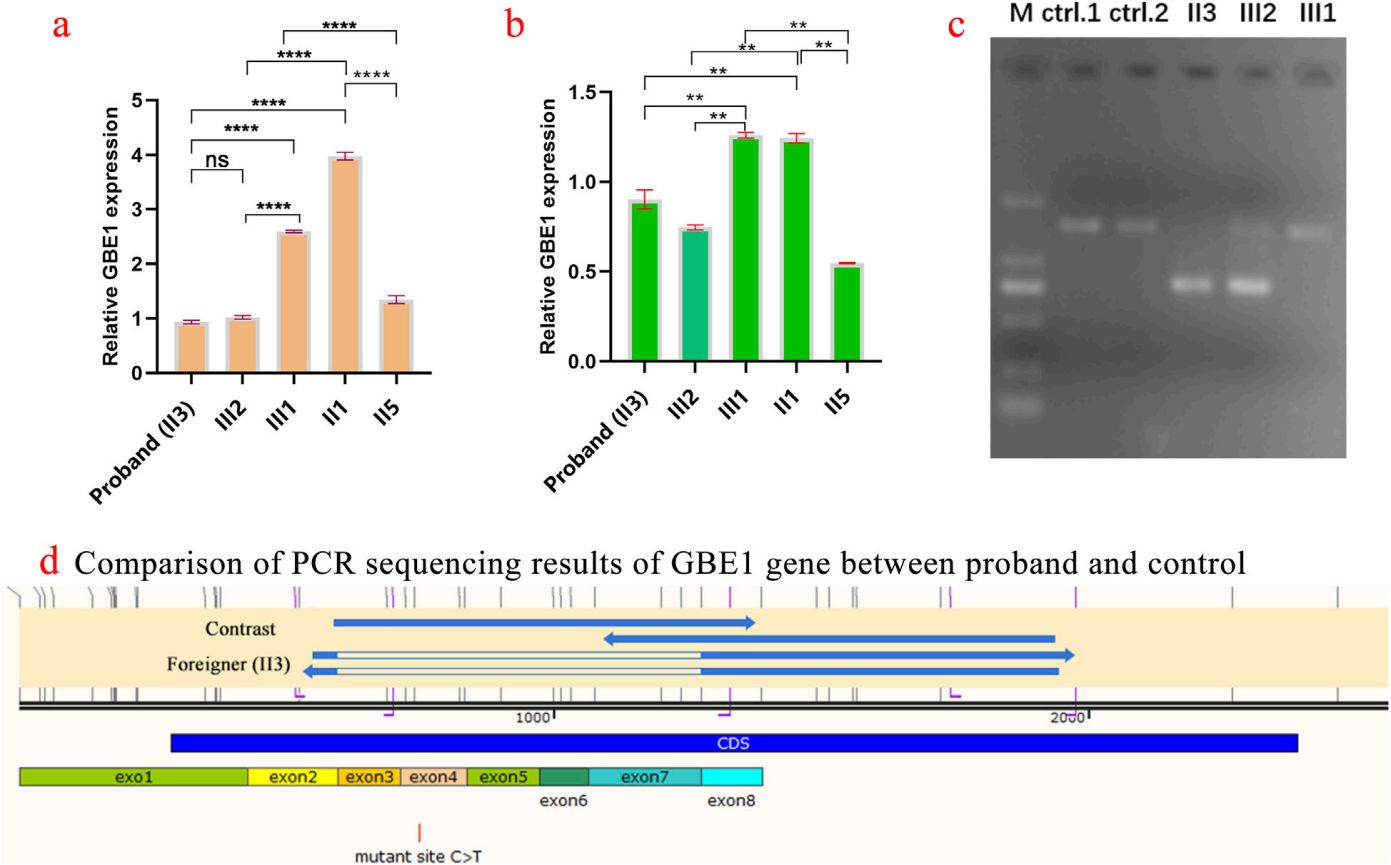
Analysis of *GBE1* gene expression and pathogenic variant in the proband and family members **(A)** Quantification of relative *GBE1* mRNA expression levels before the c.466C>T variant site in the proband (Ⅱ3) and family members (Ⅱ1, Ⅱ2, Ⅱ5) using qRT-PCR. The proband exhibits significantly lower *GBE1* mRNA levels before the pathogenic variant site compared to family members. Statistical significance is indicated by p-values (ns: not significant, *p < 0.01, **p < 0.001, ***p < 0.0001). **(B)** Quantification of relative *GBE1* mRNA expression levels after the variant site. The proband (Ⅱ3) shows significantly higher *GBE1* expression post-pathogenic variant, whereas other family members exhibit lower levels, highlighting the differential impact of the variant on *GBE1* expression. **(C)** Gel electrophoresis showing PCR products of the *GBE1* gene (expected size: 1,462 bp) from the proband (Ⅱ3), his daughter (Ⅱ1), and his brother (Ⅱ2). The gel indicates distinct band patterns among individuals, with the proband showing abnormal amplification. Control lanes (ctrl1 and ctrl2) display the expected band sizes. **(D)** PCR sequencing results of the *GBE1* gene in the proband (Ⅱ3), his son, and a healthy control. A c.466C>T (p.R156C) variant in exon 4 of *GBE1* leads to abnormal splicing in the exon 3–exon 7 region in the proband and his son, resulting in exon skipping or altered splicing. The control exhibits normal splicing.

**FIGURE 3:**
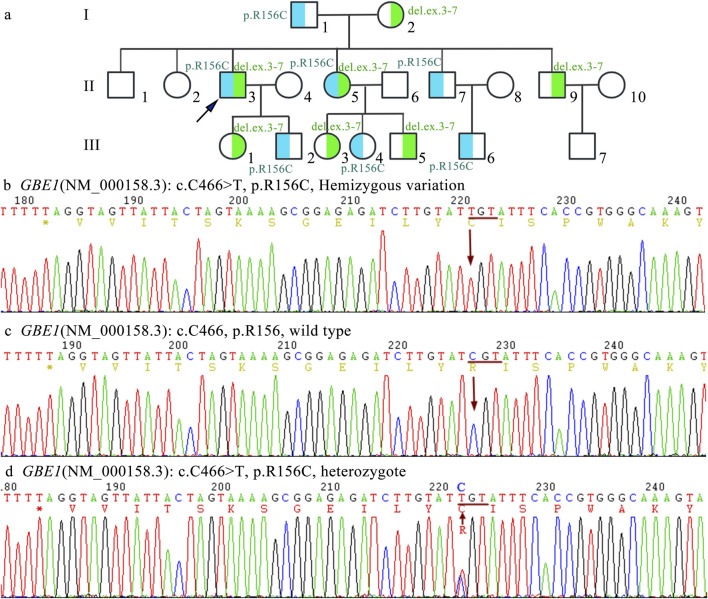
Family pedigree and Sanger sequencing analysis **(A)** The pedigree chart illustrates an autosomal recessive inheritance pattern of APBD caused by compound heterozygous pathogenic variants in the *GBE1* gene. The proband (indicated by an arrow) carries compound heterozygous variants (NM_000158.3:c.[314_992+1del];[466C>T]). The deletion variant c.314_992+1del, involving exons 3–7, is highlighted in blue, while the point mutation **(C)**466C>T (p.Arg156Cys) is highlighted in green. **(B)** Sanger sequencing results show a hemizygous mutation at position c.466, where C is replaced by T in exon 4 of the *GBE1* gene, represented as NM_000158.3:c.[466C>T];[0]. **(C)** Sanger sequencing results demonstrate the normal sequence of the wild-type allele at position c.466C (p.Arg156), represented as NM_000158.3:c.[=]. **(D)** Sanger sequencing results show the heterozygous carrier state for the variant c.466C>T (p.Arg156Cys), represented as NM_000158.3:c.[466C>T];[=].

Five family members were analyzed using primers flanking the *GBE1* variant site (c.466C>T). The mRNA levels upstream of the variant site in the proband (Ⅱ3) were significantly lower compared to his daughter (II1), brother (Ⅱ2), and sister (Ⅱ5). In contrast, the mRNA levels downstream of the *GBE1* pathogenic variant site in the proband were significantly elevated compared to the other four individuals, indicating a lower mRNA content upstream of the pathogenic variant site than downstream (Figures 2A, B). Subsequent q-PCR screening of the remaining family members revealed that the mRNA levels of the *GBE*1 pathogenic variant site, both upstream and downstream, were significantly downregulated in I1, II7, III2, III4, and III6 compared to I2, II2, II9, III1, III3, III5, and III7 (all p < 0.0001). This indicates the presence of a heterozygous deletion affecting exons 3 to 7 of the *GBE1* gene in I1, II7, III2, III4, and III6. Based on the carrier status of the c.466C>T (p.R156C) variant in the family members, it was found that the two affected patients (II3, II5) carry the compound heterozygous variant, which follows a segregated inheritance pattern (Figure 3).

### PCR amplification and sequencing

Three samples from the proband and their children were amplified using the *GBE1* PCR F/R primer, with an expected product size of 1,462 bp. The results of agarose gel electrophoresis are shown in Figure 2C. PCR sequencing confirmed abnormal splicing in the exon 3 to exon 7 region of the *GBE1* gene in the proband and their children, associated with the c.466C>T (p.R156C) variant (Figure 2D).

### Prediction and analysis of protein birth information

**FIGURE 4:**
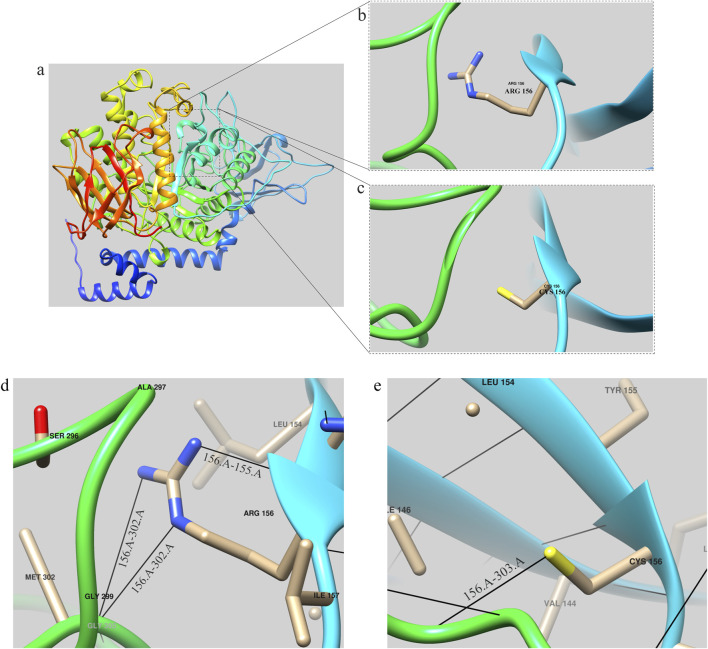
*GBE1* protein structure prediction diagram **(A)** Overall structural model of the *GBE1* protein, predicted using AlphaFold and visualized with Chimera software. **(B)** Local structural diagram highlighting the arginine residue (Arg) at position 156 in the wild-type protein. **(C)** Local structural diagram illustrating the cysteine residue (Cys) at position 156 in the mutant protein. **(D)** Visualization of hydrogen bonding interactions between Arg156 and its neighboring amino acids (155Tyr and 302Met) in the wild-type protein. **(E)** Visualization of hydrogen bonding interactions between Cys156 and its neighboring amino acids (303Phe) in the mutant protein. Note: Black lines indicate hydrogen bonds between the residues.

The tertiary structures of both the wild-type (WT) *GBE*1 protein and the c.466C>T (p.R156C) variant were predicted using AlphaFold (https://alphafold.ebi.ac.uk/entry/Q14524), as illustrated in the accompanying figures. Structural analysis indicated that the mutant protein exhibits a frameshift, resulting in the deletion of 577 amino acids present in the wild-type protein, along with the insertion of 32 new amino acids that cause the frameshift pathogenic variant. The *GBE1* protein sequence was obtained from AlphaFold (https://alphafold.com/) and visualized using Chimera software (Pettersen et al., 2004). The three-dimensional structure of the *GBE1* protein is depicted in Figure 4A.

Local structures of the arginine (Arg) and cysteine (Cys) residues at position 156 are visualized in Figures 4B, C, respectively. Hydrogen bonding interactions analyzed with Chimera software showed that the 156Arg residue in the wild-type protein formed hydrogen bonds with 155Tyr and 302Met (Figure 4D), while the 156Cys residue in the mutant formed a hydrogen bond with 303Phe (Figure 4E).

**FIGURE 5:**
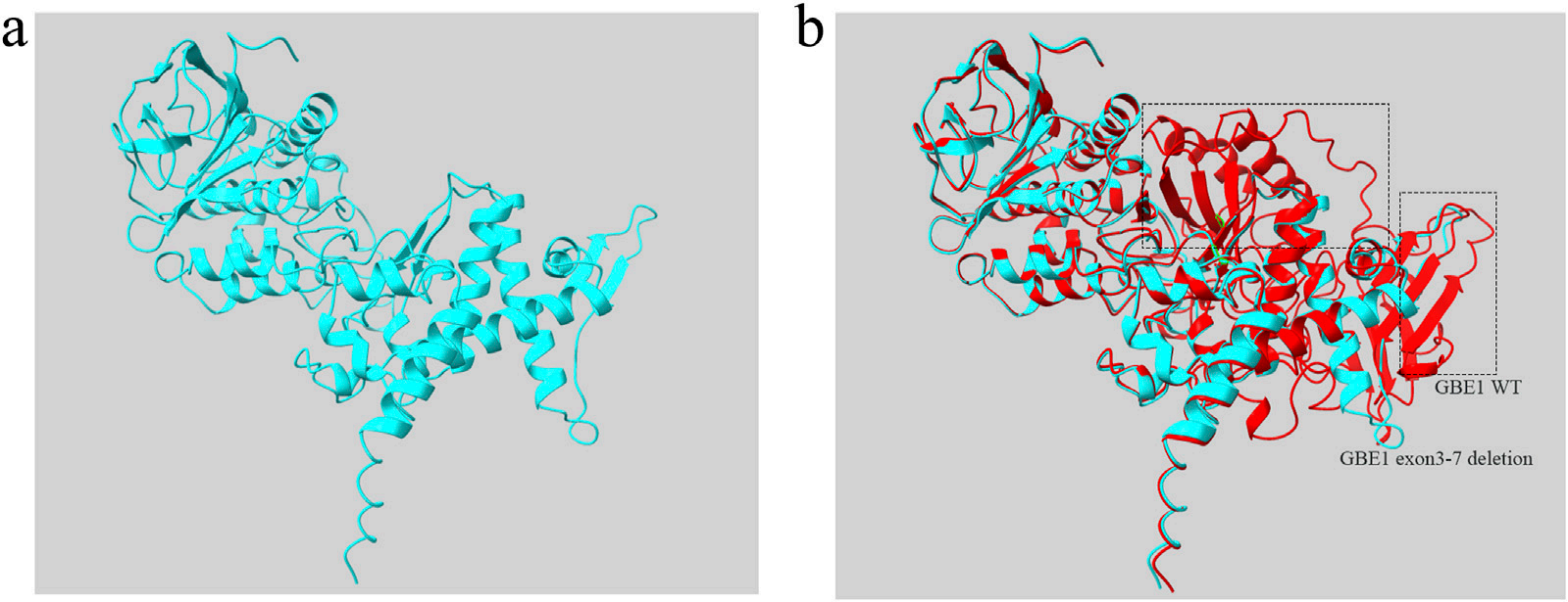
Protein structure diagram of *GBE1* exon 3–7 deletion variant **(A)** The predicted three-dimensional structure of the *GBE1* protein with the exon 3–7 deletion pathogenic variant, generated using AlphaFold and visualized with Chimera software. **(B)** Structural comparison between the *GBE1* exon 3–7 deletion mutant protein (blue) and the wild-type *GBE1* protein (red). The non-overlapping regions, indicating structural differences between the two proteins, are highlighted within the black box.

To further analyze the mutant, the sequence of the *GBE1* protein with the deletion of exons 3–7 was uploaded to the AlphaFold Server (https://alphafoldserver.com/) for structure prediction. The resultant mutant protein, comprising 475 amino acids, is shown in Figure 5A. Using Chimera software, a structural comparison between the exon 3–7 deletion mutant protein and the wild-type *GBE1* protein was performed. The root-mean-square deviation (RMSD) between 452 aligned atom pairs was 0.436 Å, indicating a high structural similarity. However, across all 475 atom pairs, the RMSD increased to 6.820 Å, reflecting the structural differences introduced by the variant (Figure 5B).

## Discussion

In this study, we describe a novel compound heterozygous variant of APBD in a family, which has not been previously reported in the literature. The patient (II3) carries a compound heterozygous variant in the *GBE1* gene, including c.466C>T (p.R156C) and a deletion of exons 3–7. Pathogenic variants in the *GBE1* gene are linked to both APBD and Glycogen Storage Disease type IV (GSD-IV), both autosomal recessive disorders. Pathogenic variations must affect both alleles simultaneously to manifest the disease (Williams and Clementson, 1991). In the proband, the combination of these two variants results in APBD (Koch et al., 2023).

The proband presents with spastic paraplegia, axonal neuropathy, and neurogenic bladder dysfunction, experiencing numbness in both lower limbs, difficulty walking, and leg lifting. Cognitive function remains relatively intact, with mild cognitive impairment noted in memory. MRI scans revealed progressive white matter deterioration near the lateral ventricles and atrophy in the lateral frontal and parietal lobes. Atrophy was also observed in the cerebral cortex and cerebellum, along with a reduction in the size of the medulla oblongata and spinal cord. Similarly, the proband’s sister exhibited movement difficulties in the lower limbs, with MRI findings showing atrophy in the cerebral cortex, cerebellum, and spinal cord, along with symmetrical white matter changes.

Most APBD patients seek medical attention in their fifties or sixties. However, no conclusive evidence links specific *GBE1* pathogenic variants to clinical severity (Williams and Clementson, 1991). After more than a decade of disease progression, many patients lose the ability to walk independently and require mobility assistance (Mochel et al., 2012). The proband began exhibiting symptoms at age 49, and after 8 years of rapid progression, now uses a wheelchair. This case represents a rapid progression subtype.

In a 2004 study by Bruno et al., two sisters with deletions in these exons died in infancy (Bruno et al., 2004). Similarly, a 2012 case by Li et al. reported a patient with a deletion of exons 2–7 who died shortly after birth due to cardiopulmonary complications (Li et al., 2012). GSD-IV, a congenital form of the disease, is often fatal in infancy (Maruyama et al., 2004). Unlike these cases, our proband exhibited a delayed onset of symptoms around age 50, with slow disease progression. Despite a deletion near this pathogenic region, the patient avoided early mortality but showed predominant nervous system and central nervous system involvement.

The combination of the *GBE1* exon 3–7 deletion and the p.R156C pathogenic variant likely contributes to the delayed onset and slow progression observed in this case (Lossos et al., 1998). APBD remains a rare disease with only around 200 confirmed cases worldwide. Due to the limited knowledge of APBD among healthcare providers, the condition is often misdiagnosed or undiagnosed, leading to an underestimation of its prevalence (Kakhlon et al., 2021; Williams and Clementson, 1991). APBD is commonly confused with conditions such as cerebrovascular disease, multiple sclerosis, prostate disease, and primary urinary system dysfunction. Survey data indicate that diagnosis can be delayed by up to 6.8 years on average, primarily due to a lack of awareness of APBD’s clinical and imaging features.

Accurate diagnosis of APBD should prioritize genomic testing, particularly whole-exome sequencing to identify pathogenic variants. Variants such as c.466C>T in *GBE1* reduce glycogen branching enzyme 1 activity, leading to the deposition of polyglucosan bodies (PB) in tissues. Detection of PB can be accomplished through sural nerve biopsy and ultrastructural examination (Chen et al., 2024). Early diagnosis is crucial to avoid unnecessary surgeries and inappropriate drug treatments. Bladder dysfunction, often occurring years before walking difficulties, is common in male patients and is frequently misdiagnosed as prostate disease, leading to unnecessary procedures. Even in the absence of white matter abnormalities, symptoms may be associated with atrophy of the medulla oblongata or spinal cord. Any adult with neurogenic bladder, spastic paraplegia, and spinal cord atrophy should be evaluated for APBD (Mochel et al., 2012; Hellmann et al., 2015; Martinez Corrales et al., 1992; Massa et al., 2008).

Individualized treatment, including antispasmodic medications, massage, ultrasound therapy, and daily exercises, can help alleviate symptoms and prevent complications such as joint contractures. For bladder dysfunction, anticholinergic drugs, catheterization, or indwelling bladder catheters may be used to prevent infection. Cognitive impairment can be addressed with pharmacological treatment and psychological support, while training in daily activities improves patient independence. Symptoms should be managed according to relevant clinical guidelines (Akman et al., 1993). Ongoing efforts are being made to develop alternative treatments for GSD-IV, including enzyme and gene replacement therapies for other GSD subtypes and neuromuscular disorders. However, the safety and efficacy of gene therapy face several challenges, such as the balance between therapeutic dosage and immune response, as well as potential toxicity associated with the delivery system. Restoring GBE enzyme activity is one of the anticipated therapeutic strategies, alongside other approaches such as reducing glycogen synthesis or enhancing lysosomal degradation of glycogen (Koeberl et al., 2024; Koch et al., 2023; Gumusgoz et al., 2022; Yi et al., 2016; Gumusgoz et al., 2021). The treatment of this disease holds promising prospects for the future. With technological advancements, it will offer more patients the potential for a cure.

## Conclusion

Previous studies have extensively explored the genetic mechanisms, clinical features, and treatment strategies for APBD, yielding significant insights. The patient presented in this report is a rare case of a compound heterozygote with familial aggregation. We identified a pathogenic variant in the *GBE1* gene, and our research contributes to the existing clinical data, offering clinicians improved diagnostic approaches and methodologies for managing rare diseases.

## Data Availability

The data underlying the results presented in the study are available from National Center for Biotechnology Information: SCV005849587 (https://www.ncbi.nlm.nih.gov/clinvar/variation/3767255/); SCV005687769 (https://www.ncbi.nlm.nih.gov/clinvar/variation/989810/).
