# Supplementary material for: Clinical genetic analysis of an adult polyglucosan body disease (APBD) family caused by the compound heterozygous variant of GBE1 p.R156C and deletion exon 3-7
Source: Front Genet. 2025 Mar 19;16:1514610. doi: 10.3389/fgene.2025.1514610 (PMC11961938; doi:10.3389/fgene.2025.1514610)
Supplement: Supplementary file 1 [file DataSheet1.docx]

Supplementary Table S1

**Supplementary Table S1 Detailed results of electromyography of the proband (Ⅱ3)**

| **Motor nerve conduction study** | | | | |
| --- | --- | --- | --- | --- |
| Nerve | Latency (ms) | Amplitude (mV) | Conduction velocity (m/s) | F-M latency (ms) |
| Ulnar nerve, motor, left | | | | |
| Wrist-ADM | 2.2 | 11.6 |  |  |
| Bl.elbow-wrist | 6.3 | 10.7 | 53.7 |  |
| Ab.elbow-Bl.elbow | 7.1 | 10.1 | 61.3 |  |
| Ulnar nerve, motor, right | | | | |
| Wrist-ADM | 2.2 | 12.5 |  |  |
| Bl.elbow-wrist | 6.7 | 11.6 | 48.9 |  |
| Ab.elbow-Bl.elbow | 8.9 | 11.3 | 50.5 |  |
| Median nerve, motor, left | | | | |
| Wrist-APB | 3.3 | 12.4 |  | 24.3 |
| Elb-wrist | 7.6 | 12.0 | 55.8 |  |
| Median nerve, motor, right | | | | |
| Wrist-APB | 4.0 | 15.1 |  | 25.2 |
| Elb-wrist | 8.2 | 14.8 | 58.3 |  |
| Tibial nerve, motor, left | | | | |
| Ankle-AHB | 4.3 | 0.3 |  |  |
| Knee-Ankle | 14.7 | 0.3 | 38.5 |  |
| Tibial nerve, motor, right | | | | |
| Ankle-AHB | 4.4 | 0.7 |  |  |
| Knee-Ankle | 14.2 | 0.7 | 38.8 |  |
| Peroneal nerve, motor, left | | | | |
| Ankle-EDB | 5.0 | 0.5 |  |  |
| Be knee-Ankle | 13.0 | 0.4 | 37.5 |  |
| Ab knee-Be knee | 14.3 | 0.4 | 48.2 |  |
| -Ab knee | 3.1 | 3.8 |  |  |
| Peroneal nerve, motor, right | | | | |
| Ankle-EDB | 4.4 | 0.6 |  |  |
| Be knee-Ankle | 12.7 | 0.5 | 37.3 |  |
| Ab knee-Be knee | 13.8 | 0.5 | 48.2 |  |
| -Ab knee | 2.2 | 4.3 |  |  |
| **Sensory nerve conduction study** | | | | |
| Nerve | Peak latency (ms) | Amplitude (uV) | Conduction velocity (m/s) | |
| Ulnar nerve, feeling, left | | | | |
| Dig Ⅴ- wrist | 2.0 | 11.0 | 49.0 | |
| Ulnar nerve, feeling, right | | | | |
| Dig Ⅴ- wrist | 2.0 | 9.4 | 47.5 | |
| Median nerve, feeling, left | | | | |
| Dig Ⅰ-wrist | 2.1 | 14 | 51.4 | |
| Dig Ⅲ-wrist | 2.4 | 15 | 56.2 | |
| Median nerve, feeling, right | | | | |
| Dig Ⅰ-wrist | 2.3 | 8.8 | 47.8 | |
| Dig Ⅲ-wrist | 2.4 | 10 | 50.8 | |
| Peroneal nerve, feeling, left | | | | |
| Ankle-Foreleg | 2.0 | 11.0 | 49.0 | |
| Peroneal nerve, feeling, right | | | | |
| Ankle-Foreleg | 2.0 | 9.4 | 54.2 | |
| Sural nerve, feeling, right | | | | |
| Ankle-Foreleg | 3.2 | 2.9 | 45.3 | |
| Sural nerve, feeling, left | | | | |
| Ankle-Foreleg | 2.7 | 3.2 | 45.6 | |
